# Post-transplant Alternative Complement Pathway Activation Influences Kidney Allograft Function

**DOI:** 10.1007/s00005-019-00541-w

**Published:** 2019-04-26

**Authors:** Dorota Bartoszek, Oktawia Mazanowska, Katarzyna Kościelska-Kasprzak, Agnieszka Lepiesza, Marta Myszka, Marcelina Żabińska, Magdalena Krajewska, Marian Klinger

**Affiliations:** 1 Department of Nephrology and Transplantation Medicine, Faculty of Postgraduate Medical Training, Wroclaw Medical University, Borowska 213, 50-556 Wrocław, Poland; 2 Faculty of Dentistry, Wroclaw Medical University, Krakowska 26, 50-425 Wrocław, Poland; 3 Department of Vascular, General and Transplant Surgery, Faculty of Postgraduate Medical Training, Wroclaw Medical University, Borowska 213, 50-556 Wrocław, Poland

**Keywords:** Alternative pathway, Functional complement activity, Kidney transplantation, Kidney recipients

## Abstract

The complement system is one of the crucial pathophysiological mechanisms that directly influence the function of a transplanted kidney. Since the complement pathways’ activation potential can be easily determined via their functional activity measurement, we focused on fluctuation in the cascade activity in the early post-transplant period. The aim of the study was to relate the kidney transplantation-induced complement system response to allograft outcome. Forty-two kidney recipients (aged: 53.5 [37–52], 17 females/25 males) and 24 healthy controls (aged: 40.5 [34–51], 13 females/11 males) were enrolled in the study. The functional activities of alternative, classical, and lectin pathways were determined before and in the first week after transplantation using Wielisa^®^-kit. We observed that the baseline functional activity of the alternative pathway (AP) was higher in chronic kidney disease patients awaiting transplantation compared to healthy controls and that its level depended on the type of dialysis. AP-functional activity was decreased following transplantation procedure and its post-transplant level was related to allograft function. The baseline and transplantation-induced functional activities of the classical and lectin pathways were not influenced by dialysis type and were not associated with transplant outcome. Moreover, our study showed that intraoperative graft surface cooling had a protective effect on AP activation. Our study confirms the influence of dialysis modality on persistent AP complement activation and supports the role of AP in an early phase after kidney transplantation and allograft outcome.

## Introduction

The complement system, a major player of innate immunity, is considered one of the crucial pathophysiological mechanisms directly influencing the function of a transplanted kidney. Complement activation has been highlighted in various aspects of renal transplantation including graft quality, ischemia–reperfusion injury, and cellular or antibody-mediated rejection (Jager et al. 2017).

The complement system consists of over 40 soluble and membrane-bound components, regulators and receptors. Their major function is to recognize and eliminate pathogens, but they are also involved in removal of immune complexes and apoptotic cells from the circulation and tissues. Complement cascade activation is also initiated by metabolic and physical changes in the body. The complement proteins are sequentially arranged in the three pathways of activation: classical (CP), lectin (LP), and alternative (AP) pathways (Kościelska-Kasprzak et al. 2014). Independently of the mechanism, activation of any of the three pathways generates C3 and C5 convertase, and subsequently releases C3a and C5a anaphylatoxins. In the final stage of complement activation, C5b–9 membrane attack complex is formed, leading to cell membrane damage and cell lysis. The complex is also capable of activating neutrophils, endothelium, and epithelium (Bajic et al. 2015).

The classical pathway is initiated by antigen–antibody immune complexes binding to C1q, a component of C1 complex. C1q, after binding to the Fc region of IgG or IgM, initiates the release of serine proteases C1r and C1s and further activation of complement cascade. Activation of the lectin pathway depends on mannose-binding lectin (MBL), ficolins or collectin-11, which attach to carbohydrate ligands on the surface of microorganisms. The AP is constantly self-activated by spontaneous hydrolysis of C3–C3(H_2_O) and strictly regulated by membrane-bound regulators. Downregulation of the expression of complement regulators (Thurman et al. 2006) or upregulation of the local production of complement components (Farrar et al. 2006; Pratt et al. 2002) is suggested to be responsible for ischemia–reperfusion injury of tubular epithelial cells during kidney transplantation (KTx).

Since the potential of complement pathways’ activation can be easily determined via their functional activity measurement, we focused on induced fluctuation in the cascade activity in the early post-transplantation period. The aim of the study was to relate the KTx-induced complement system to short-term allograft outcome so as to validate its usefulness in immune monitoring of renal transplant recipients.

## Materials and Methods

Forty-two renal transplant recipients (all Caucasians, 17 females/25 males, aged: 25–75) who underwent transplantation between 2012 and 2015 at Wroclaw Medical University, Poland, were enrolled in this study. Renal allografts were received from 25 deceased donors (6 females/19 males, aged: 19–70). Seven patients received a second renal transplant. Forty (95%) recipients were treated with dialysis, 34 (85%) with hemodialysis and six (15%) with peritoneal dialysis. In the study group, 26 (62%) recipients were operated on with kidney surface cooling during the time of implantation. In those cases, warm ischemia was avoided using a specially designed disposable polyethylene bag produced by Raguse GmbH (Germany). The polyethylene bag was removed from the transplanted kidney immediately after completion of the vascular anastomoses followed by graft reperfusion. The other recipients were operated on with a standard technique. Four patients presented pre-transplant panel reactive antibodies (PRA) of above 20%. The initial immunosuppressive therapy consisted of tacrolimus, mycophenolate mofetil/sodium and prednisone in 31 cases or cyclosporine A, mycophenolate mofetil/sodium, and prednisone in 11 cases. The recipients were followed up for 3 months after transplantation. The general characteristics of kidney transplant recipients and deceased donors are summarized in Table 1. In addition, a healthy control group (24 volunteers, 13 females/11 males, aged: 24–65) with no clinical history of kidney diseases was included in the study.

**Table 1:** Characteristics of the study groups

Recipients	Number (%) or median [IQR]
Gender (female/male)	17 (40%)/25 (60%)
Age (years)	53.5 [37–52]
Cause of renal failure	
Chronic glomerulonephritis	16 (38%)
Diabetic nephropathy	2 (5%)
Hypertensive nephropathy	8 (19%)
Polycystic renal disease	5 (12%)
Other/unknown	11 (26%)
Retransplant	7 (17%)
HLA A mismatch (0/1/2)	3/23/16
HLA B mismatch (0/1/2)	3/20/19
HLA DR mismatch (0/1/2)	12/19/11
Max PRA > 20%	6 (14%)
Last PRA > 20%	4 (12%)
Time on dialysis before KTx (months)	20.8 [16–38]
Dialysis modality HD/PD	34/6
DGF	8 (19%)
AR	5 (12%)
Initial immunosuppression	
TAC + MPA + steroids	31 (74%)
CsA + MPA + steroids	11 (26%)
Donors	
Gender (female/male)	6 (24%)/19 (76%)
Age (years)	37.5 [29–56]
CIT (h)	27 [24–32]
WIT > 0	16 (38%)
WIT = 0	26 (62%)
Control group	
Gender (female/male)	13 (54%)/11 (46%)
Age (years)	40.5 [34–51]

*HD* hemodialysis, *PD* peritoneal dialysis, *PRA* panel reactive antibodies, *DGF* delayed graft function, *AR* acute rejection, *KTx* kidney transplantation, *CIT* cold ischemia time, *WIT* warm ischemia time

Five blood samples from each kidney allograft recipient were collected: pre-transplant (prior to the initiation of immunosuppression), 1 h after reperfusion, and 1, 3, and 7 days after transplantation. Venous blood was drawn into the serum separator tubes. Samples were processed immediately according to the standard laboratory protocols. After coagulation at room temperature, samples were centrifuged at 1000×*g* for 15 min and the serum was collected and stored at − 80 °C until the day of assessment.

The three functional complement pathways’ activation potential was measured using a commercially available ELISA test (Wielisa^®^-kit COMP 300, Sweden) according to the manufacturer’s instruction. The functional complement activity for each pathway was expressed as a percentage of activity obtained for the manufacturer positive control serum. Reference normal level was 100%. In all cases, decreased functional activity was assumed to reflect the activation status of the pathway.

Estimated glomerular filtration rate (eGFR) was calculated according to the modification of diet in renal disease study formula (Levey et al. 1999).

The study was approved by the Bioethics Committee of the Medical University in Wroclaw, Poland, in accordance with the World Medical Association Declaration of Helsinki—Ethical Principles for Medical Research Involving Human Subjects. All patients provided their fully informed consent before participation in the study.

### Statistical Analysis

All statistical analyses were performed using Statistica 12.0 (StatSoft, Poland). The normality of data distribution was tested with Shapiro–Wilk test. The numerical data were presented as mean ± SD for normally distributed variables and median, interquartile range for the others. As the functional complement activity was not normally distributed, non-parametric Mann–Whitney *U* test, Wilcoxon test, and Spearman correlation were used for data analysis. Differences between the levels of functional complement activity at various timepoints were assessed by Wilcoxon paired test. *p* value below 0.05 was considered statistically significant.

## Results

### Functional Complement Activity in the Study Groups

Functional complement activity in healthy volunteers was AP_HC_ 73% [62–88], CP_HC_ 112% [104–116], and LP_HC_ 85% [40–110]. The observed values were within the normal range as specified by the manufacturer.

In renal transplant recipients, the functional complement activity was assessed directly before KTx, up to 1 h after reperfusion and 1, 3, and 7 days after transplantation. The results are summarized in Table 2.

**Table 2:** Functional activity of the three complement pathways observed for renal transplant recipients and healthy controls

Pathway	Control group	Time since Ktx	Recipients group	Comparison of KTx with HC (*p* value)*	Comparison with pre-KTx value (*p* value)*
	Functional complement activity [%] Median, IQR		Functional complement activity [%] Median, IQR		
Alternative pathway	73 [62–88]	Pre-KTx	101 [78–117]	**0.001**	–
		1 h	99 [71–107]	**0.021**	**0.044**
		24 h	88 [63–108]	0.138	0.056
		3 days	92 [76–115]	**0.005**	0.802
		7 days	109 [75–133]	**< 0.001**	0.265
Classical pathway	112 [104–116]	Pre-KTx	102 [97–112]	**0.029**	–
		1 h	101 [92–107]	**< 0.001**	**0.002**
		24 h	105 [98–113]	**0.034**	0.984
		3 days	114 [104–122]	0.393	**0.004**
		7 days	109 [101–116]	0.446	0.253
Lectin pathway	85 [40–110]	Pre-KTx	97 [89–116]	0.131	–
		1 h	93 [75–108]	0.529	0.071
		24 h	94 [66–106]	0.817	**0.016**
		3 days	96 [64–114]	0.454	0.384
		7 days	99 [48–113]	0.547	0.379

*Statistically significant *p* values are marked in bold

Pre-transplant level of AP-functional activity was higher compared to healthy controls (AP_preKTx_ 101% [78–117] vs AP_HC_ 73% [62–88], *p *= 0.001). Kidney transplantation-induced decrease in AP-functional activity that proved significant 1 h after reperfusion (AP_1h_ 99% [71–107], *p *= 0.044). AP-functional activity 24 h and 3 days after transplantation was similar to the level observed as soon as 1 h after transplantation (AP_24h_ 88% [63–108], *p *= 0.682 and AP_3d_ 99% [71–107], *p *= 0.058). Seven days after transplantation, the significant increase in AP-functional activity from a dropdown level measured 1 h after reperfusion was observed (AP_7d_ 109% [75–133], *p *= 0.004). AP-functional activity 1 week after transplantation was again significantly elevated when compared to healthy control group (*p *< 0.001).

Contrary to AP, pre-transplant CP functional activity was lower compared to healthy controls (CP_preKTx_ 102% [97–112], CP_HC_ 112% [104–116], *p *= 0.029). After KTx, we observed a slight decrease in CP functional activity 1 h after reperfusion (CP_1h_ 101% [92–107], *p *= 0.002) and its subsequent significant increase to the value of 105% [98–113] observed 24 h after KTx (*p *= 0.003). The CP_24h_ functional activity was similar to pre-transplant level (*p *= 0.984). After 3 days, CP functional activity increased further and reached the values observed for healthy controls (CP_3d_ 114% [104–122], *p *= 0.393).

Pre-transplant LP functional activity was similar to healthy controls (LP_preKTx_ 97% [89–116], LP_HC_ 85% [40–110], *p *= 0.131) and it was generally not influenced by KTx, except for a slight decrease observed after 24 h (LP_24h_ 94% [66–106], *p *= 0.016). The observed values were similar to healthy controls at each timepoint (*p *> 0.05).

### Clinical Factors Influencing Functional Complement Activity

We analyzed the association of the recipients’ clinical and demographic characteristics with the observed functional complement activity. The functional complement activity was not related to recipient age, gender, PRA status, or initial immunosuppressive therapy.

We observed that AP-functional activity was not only elevated in patients compared to healthy controls, but it was also influenced by pre-transplant dialysis type. In peritoneal dialysis treated patients, AP was significantly higher than in the patients treated with hemodialysis (PD AP_preKTx_ 135% [129–143], HD AP_preKTx_ 93% [75–107], *p *< 0.001). Both peritoneal (*p *< 0.001) and hemodialysis (*p *= 0.023)-treated patients presented higher levels of AP-functional activity compared to healthy controls (AP_HC_ 73% [62–88]).

Classical and lectin pathways were not affected by dialysis modality. In addition, no significant correlation was observed between any pathway functional complement activity and time on dialysis.

When the peri-surgical parameters were analyzed, we observed that functional complement activity was influenced by warm ischemia. In particular, the recipients operated on with graft surface cooling presented significantly higher level of AP-functional complement activity one and 24 h after reperfusion (AP_1h_ 105% [97–114], AP_24h_ 103% [74–127]) than patients who were operated on with standard procedures (AP_1h_ 74% [52–99], *p *= 0.006 and AP_24h_ 68% [47–89], *p *= 0.002). Cold ischemia time was not associated with the first week functional complement activity.

### The Influence of Complement Activity on Kidney Graft Function

We observed positive correlations between AP-functional activity and short-term allograft outcome. Functional complement activity of AP measured 3 days after transplantation was significantly lower in the recipients experiencing a delayed graft function (DGF; AP_3d_ 71% [59–95]) compared to those with immediate graft function (AP_3d_ 96% [84–117], *p *= 0.047).

We also observed that post-transplant glomerular filtration rate was associated with AP-functional activity 3 days after KTx (3rd day eGFR *r* = 0.36, *p *= 0.019; 7th day eGFR *r* = 0.39, *p *= 0.010; 3rd month eGFR *r* = 0.40, *p *= 0.042).

CP and LP functional activities were not related to graft function.

## Discussion

In recent years, considerable progress has been made in understanding the role of complement cascade in various aspects of KTx-induced immune activation. However, neither the significance of the complement system nor its mode of action in the peri-transplant period and its impact on transplant outcome have been fully elucidated (Cernoch and Viklicky 2017; Damman et al. 2008; Farrar and Sacks 2014; Jager et al. 2017).

We aimed to study the transplant-induced activation of the complement pathways by a bench-to-bedside measure. We focused on the pathway directed assessment of functional complement activity, which is an easily applicable assay that provides a concurrent description of the reservoirs of components for each of the activation pathways. When evaluated consecutively, the assay could highlight the engaged pathway and weigh the level of its activation (Palarasah et al. 2011; Seelen et al. 2005).

We observed that the baseline functional activity of AP is higher in the end-stage renal disease patients awaiting transplantation than in healthy controls. Inoshita et al. (2010) observed a dialysis-related increase in activity of all three complement pathways in a general population of HD patients compared to healthy controls. In our case, the study group included selected dialysis patients from the kidney transplant waiting list. An increased complement activity in dialysis patients could be caused by many diseases related to aging and accompanying morbidities (Poppelaars et al. 2018). The increase in activity of only AP observed in case of our kidney transplant waiting list patients could be related to their better general condition compared to the other dialyzed patients. We also noted that its level depended on the type of dialysis with increased values related to peritoneal dialysis.

End-stage renal disease leads to reduced elimination of factor D, which is a serine protease responsible for a rate-limiting step of AP of complement activation (Reddingius et al. 1993). This phenomenon may underlie the observed higher functional activity of AP in dialysis patients. Due to bioincompatibility of HD membranes and PD fluids, AP is systemically activated during HD, while PD leads to local complement activation. As a result, systemically assessed AP-functional activity is decreased in HD patients compared to PD ones.

Despite significant progress in biocompatibility of hemodialysis membranes and peritoneal dialysis fluids, complement activation remains an undesired effect of the therapy. It directly stimulates inflammation and coagulation, and also promotes infections, fibrosis, and cardiovascular events (Poppelaars et al. 2018).

Kidney transplantation is associated with donor death related graft injury, ischemia–reperfusion injury, and immune allostimulation. Ischemia–reperfusion injury results in locally triggered activation of AP in the kidney. We observed the transplantation-induced systemic decrease in AP-functional activity that proved significant 1 h after reperfusion (*p *= 0.044) and was sustained until the third day after KTx. The level of ischemia–reperfusion-related AP activation is probably sufficient to lead to systemically visible change in AP-functional activity. While the trigger is no longer present, AP-functional activity returns to its baseline level defined by a higher factor D concentration.

Our results are in accordance with experimental studies which confirmed AP involvement in ischemia–reperfusion injury. A contribution of the complement system as a response to ischemia–reperfusion on tissue injury has been demonstrated in various animal models (Casiraghi et al. 2017; Moller-Kristensen et al. 2005; Pratt et al. 2000; Zhou et al. 2000, 2006). Zhou et al. (2000) demonstrated that C3^−/−^, C5^−/−^, and C6^−/−^ deficient mice were protected from renal ischemia–reperfusion injury, while C4^−/−^ deficient mice were not. The complement system is immediately activated after ischemia–reperfusion and complement components are present on renal tubules as soon as after 60 min, with peak activation after 24 h (Farrar et al. 2004).

Most of the complement components are produced by the liver. However, it was shown that complement proteins can also be produced by renal tissue, T cells, and antigen-presenting cells (APCs). All cellular compartments of the kidney are able to synthesize complement proteins, but tubuloepithelial cells are the major source of local complement production (Montero et al. 2016). Although the alternative pathway activation occurs probably mainly due to the local release of C3 component in renal tissue (Pratt et al. 2000), we were able to note the post-transplant systemic activation of the pathway.

Some data also suggest the involvement of MBL and pattern recognition of endothelial cell surface ligands exposed due to the ischemia, acting via standard MBL pathway or directly influencing the alternative one without the need for C4 activation (Moller-Kristensen et al. 2005). The results of our study did not show involvement of LP in the first week after transplantation.

None of the previously published work measured functional complement activity of the particular pathways in the sera of kidney recipients or assessed their influence on kidney graft function. We demonstrated a positive correlation between the third post-transplant day AP-functional activity and glomerular filtration up to 3 month post-KTx. The positive correlation suggests that lower AP activation (reflected in higher functional activity measurement) is related to better allograft function; however, CP- and LP-functional activity was not related to graft function. In addition, the decrease in AP-functional activity was related to DGF. Błogowski et al. (2012) suggested that perioperative level of sC5b–9/membrane attack complex could be used as a potential clinical marker of post-transplant renal allograft function. In this study, the level of sC5b-9 was significantly higher in the recipients with DGF.

Moreover, our study showed a protective effect of intraoperative graft surface cooling on AP activation. The work by Kamińska et al. (2016) demonstrated that elimination of warm ischemia time had a positive impact on early allograft function and significantly diminished the DGF and rejection rate.

Activation of the complement cascade has an important role in the development of humoral and cellular alloreactivity. There are several hypotheses on the role of complement cascade in allograft rejection. C3 split products, C3b and C3d, deposited on APCs may increase antigen uptake and its presentation to T cells, which aids generation of alloreactive clones (Takada et al. 1997). C3-positive APCs (dendritic cells, macrophages, and epithelial cells) are shown to potentiate the T-cell response in vitro (Pratt et al. 2002). C3-deficient macrophages have impaired capability to stimulate T cells (Li 2004; Peng et al. 2006; Zhou et al. 2006). In addition, C3a and C5a binding to T-cell receptors may directly stimulate their alloreactivity. The antibody-mediated renal allograft rejection involves donor-specific antibodies and classical pathway of complement system activation. During its activation, C4 is cleaved and forms a bond with the cell membrane close to the site of antibody attachment. The resultant C4b is quickly degraded by proteolytic enzymes, leaving C4d strongly attached to the cell membrane. The deposition of C4d in renal graft is strongly related to antibody-mediated rejection. According to a recent report, the deposition of covalently linked C4d on erythrocytes was more strongly related to histological rejection signs than PTC-C4d staining, but this observation needs to be confirmed in further studies (Haidar et al. 2012). In our study, complement activation did not impact the occurrence of renal allograft rejection, contrary to the results of experimental models (Casiraghi et al. 2017; Lin et al. 2006; Pratt et al. 2002), most probably due to a small number of acute rejection episodes in the study group.

In summary, our study supports the role of complement AP in the early phase after KTx. We demonstrated the relationship between AP-functional activity and graft function. Renal transplant recipients who developed DGF had significantly lower level of AP-functional complement activity 3 days after transplantation. Moreover, post-transplant eGFR was associated with AP-functional activity 3 days after KTx (3rd day eGFR *r* = 0.36, *p *= 0.019; 7th day eGFR *r* = 0.39, *p *= 0.010; 3rd month eGFR *r* = 0.40, *p *= 0.042). Our results also confirm the influence of dialysis modality on persistent complement activation.

## Abbreviations

AP: Alternative pathway
APCs: Antigen-presenting cells
CP: Classical pathway
DGF: Delayed graft function
eGFR: Estimated glomerular filtration rate
HD: Hemodialysis
KTx: Kidney transplantation
LP: Lectin pathway
PD: Peritoneal dialysis
PRA: Panel reactive antibodies

## References

[CR1] Bajic G, Degn SE, Thiel S (2015). Complement activation, regulation, and molecular basis for complement-related diseases. EMBO J.

[CR2] Błogowski W, Dołegowska B, Sałata D (2012). Clinical analysis of perioperative complement activity during ischemia/reperfusion injury following renal transplantation. Clin J Am Soc Nephrol.

[CR3] Casiraghi F, Azzollini N, Todeschini M (2017). Complement alternative pathway deficiency in recipients protects kidney allograft from ischemia/reperfusion injury and alloreactive T cell response. Am J Transpl.

[CR4] Cernoch M, Viklicky O (2017). Complement in kidney transplantation. Front Med.

[CR5] Damman J, Schuurs TA, Ploeg RJ (2008). Complement and renal transplantation: from donor to recipient. Transplantation.

[CR6] Farrar CA, Sacks SH (2014). Mechanisms of rejection: role of complement. Curr Opin Organ Transpl.

[CR7] Farrar CA, Wang Y, Sacks SH (2004). Independent pathways of P-selectin and complement-mediated renal ischemia/reperfusion injury. Am J Pathol.

[CR8] Farrar CA, Zhou W, Lin T, Sacks SH (2006). Local extravascular pool of C3 is a determinant of postischemic acute renal failure. FASEB J.

[CR9] Haidar F, Kisserli A, Tabary T (2012). Comparison of C4d detection on erythrocytes and PTC-C4d to histological signs of antibody-mediated rejection in kidney transplantation. Am J Transpl.

[CR10] Inoshita H, Ohsawa I, Kusaba G (2010). Complement in patients receiving maintenance hemodialysis: functional screening and quantitative analysis. BMC Nephrol.

[CR11] Jager NM, Poppelaars F, Daha MR (2017). Complement in renal transplantation: the road to translation. Mol Immunol.

[CR12] Kamińska D, Kościelska-Kasprzak K, Chudoba P (2016). The influence of warm ischemia elimination on kidney injury during transplantation—clinical and molecular study. Sci Rep.

[CR13] Kościelska-Kasprzak K, Bartoszek D, Myszka M (2014). The complement cascade and renal disease. Arch Immunol Ther Exp.

[CR14] Levey AS, Bosch JP, Lewis JB (1999). A more accurate method to estimate glomerular filtration rate from serum creatinine: a new prediction equation. Modification of Diet in Renal Disease Study Group. Ann Intern Med.

[CR15] Li K (2004). Complement activation regulates the capacity of proximal tubular epithelial cell to stimulate alloreactive T cell response. J Am Soc Nephrol.

[CR16] Lin T, Zhou W, Farrar CA (2006). Deficiency of C4 from donor or recipient mouse fails to prevent renal allograft rejection. Am J Pathol.

[CR17] Moller-Kristensen M, Wang W, Ruseva M (2005). Mannan-binding lectin recognizes structures on ischaemic reperfused mouse kidneys and is implicated in tissue injury. Scand J Immunol.

[CR18] Montero RM, Sacks SH, Smith RA (2016). Complement—here, there and everywhere, but what about the transplanted organ?. Semin Immunol.

[CR19] Palarasah Y, Nielsen C, Sprogøe U (2011). Novel assays to assess the functional capacity of the classical, the alternative and the lectin pathways of the complement system. Clin Exp Immunol.

[CR20] Peng Q, Li K, Patel H (2006). Dendritic cell synthesis of C3 is required for full T cell activation and development of a Th1 phenotype. J Immunol.

[CR21] Poppelaars F, Faria B, Gaya da Costa M (2018). The complement system in dialysis: a forgotten story?. Front Immunol.

[CR22] Pratt JR, Abe K, Miyazaki M (2000). In situ localization of C3 synthesis in experimental acute renal allograft rejection. Am J Pathol.

[CR23] Pratt JR, Basheer SA, Sacks SH (2002). Local synthesis of complement component C3 regulates acute renal transplant rejection. Nat Med.

[CR24] Reddingius RE, Schröder CH, Daha MR (1993). The serum complement system in children on continuous ambulatory peritoneal dialysis. Perit Dial Int.

[CR25] Seelen MA, Roos A, Wieslander J (2005). Functional analysis of the classical, alternative, and MBL pathways of the complement system: standardization and validation of a simple ELISA. J Immunol Methods.

[CR26] Takada M, Nadeau KC, Shaw GD (1997). The cytokine-adhesion molecule cascade in ischemia/reperfusion injury of the rat kidney. J Clin Invest.

[CR27] Thurman JM, Ljubanović D, Royer PA (2006). Altered renal tubular expression of the complement inhibitor Crry permits complement activation after ischemia/reperfusion. J Clin Invest.

[CR28] Zhou W, Farrar CA, Abe K (2000). Predominant role for C5b-9 in renal ischemia/reperfusion injury. J Clin Invest.

[CR29] Zhou W, Patel H, Li K (2006). Macrophages from C3-deficient mice have impaired potency to stimulate alloreactive T cells. Blood.

